# Finding non-crystallographic symmetry in density maps of macromolecular structures

**DOI:** 10.1007/s10969-013-9157-7

**Published:** 2013-07-24

**Authors:** Thomas C. Terwilliger

**Affiliations:** Los Alamos National Laboratory, Bioscience Division, Los Alamos, NM 87545 USA

**Keywords:** Symmetry, Macromolecule, Crystal structure, Density map, Automation, Macromolecular crystallography, *Phenix*

## Abstract

The internal symmetry of a macromolecule is both an important aspect of its function and a useful feature in obtaining a structure by X-ray crystallography and other techniques. A method is presented for finding internal symmetry and other non-crystallographic symmetry in a structure based on patterns of density in a density map for that structure. Regions in map that are similar are identified by cutting out a sphere of density from a region that has high local variation and using an FFT-based correlation search to find other regions that match. The relationships among correlated regions are then refined to maximize their correlations and are found to accurately represent non-crystallographic symmetry in the map.

## Introduction

Non-crystallographic symmetry (NCS) is common in macromolecular crystals, occurring in about 1/3 or more of structures in the Protein Data Bank [2, 3, 24]. Though non-crystallographic symmetry increases the complexity of structure determination, in most cases it yields a distinct advantage in this process because it brings with it information on relationships between density in different parts of the crystal and between coordinates in different parts of the structure [4, 12–14]. These relationships greatly improve density modification and refinement, two key steps in structure determination.

There are several commonly used approaches for finding NCS. One is simple examination of a model that has more than one copy of a chain. This approach of course requires a model. It can be used after molecular replacement has been carried out or after a model has been built.

Another approach is to find symmetry relationships among subsets of atoms in a heavy-atom or anomalously scattering atom substructure [11, 17]. These symmetry relationships often reflect non-crystallographic symmetry, and the presence of the NCS can be checked by comparison of the density in the resulting electron density map at NCS-related positions and orientations. This approach can be used near the beginning of a SAD, MAD, or other structure determination where a substructure is obtained prior to full structure determination. Of course this approach also requires that the NCS is present in the substructure. This approach may be difficult to apply if there are only one or two atoms per NCS copy in the substructure and the NCS does not have point-group symmetry. This is because the relative orientations of the NCS-related parts of the substructure are not known in this case.

A third approach that can be applied in cases where proper NCS (e.g., a two-fold axis) is present is to search for locations in a map where nearby points related by proper symmetry have similar density [22].

A fourth and very general approach to finding non-crystallographic symmetry is to find patterns of density that are present in more than one part of an electron density map. Pai et al. [10] used a step-wise procedure of first finding possible C_α_ locations with pattern-matching techniques, classifying the locations with rotation-invariant classifiers, and then finding pairs of these locations that have similar patterns of density surrounding them. This method can be fast and effective, but has the potential limitations that the map has to be of high enough quality to locate C_α_ positions and that for maximal speed the patterns of density need to be initially represented in a rotation-invariant fashion.

Here we present an approach to finding non-crystallographic symmetry by directly searching for patterns of density that are present in more than one place in a map. This approach uses superposition of a cut-out region of the map with the remainder of the map, and therefore can make use of all the local features in a map, not just rotation-invariant ones. In this approach, an FFT-based convolution search [5, 16] is used to find orientations and translations that relate parts of a map to other parts of a map. We find that this is a rapid and accurate way to identify NCS, even in a case where the map is poor and the correlation of NCS-related density is low.

## Methods

### Locating a region that is part of the macromolecule

The approach used here to identify non-crystallographic symmetry from an electron density map has three basic parts. First, a location in the map is found that is likely to be inside the molecule. This is important as it is the local symmetry of the molecule or molecules in the crystal that are of interest, so considering a region inside the molecule is essential for success. The method used to identify such a location in the map is similar to a method commonly used to distinguish macromolecule from solvent in a map [23]. For each grid point in the electron density map, the local rms variation in electron density is calculated. This is done by simply calculating the standard deviation of the density in the map inside a sphere with radius *R* (typically 10 Å), centered at the grid point in question. Then the grid point in the map with the highest local standard deviation of density is considered the best candidate for a position inside the macromolecule. Additional points likely to be inside the macromolecule can optionally be found by choosing additional grid points with high local variation in density that are well separated (typically at least 15 Å) from previously chosen locations.

### Searching for non-crystallographic symmetry

Next, a search for NCS relating the density near the candidate point inside the macromolecule with density elsewhere in the asymmetric unit of the crystal is carried out. To make the calculation rapid, the resolution of the map used for this calculation may be chosen to be lower than the resolution of the original map (typically a resolution of 4 Å is used). A sphere of density (typically with a radius of 10 Å) is cut out of the map, centered at this position within the molecule. This sphere of density is the object that is to be compared with density everywhere in the map. Possible rotations of this sphere of density are sampled (typically each rotation differing by about 20 degrees from all others). For each rotation, the density is transformed to the new orientation, and an FFT-based convolution search [5, 16] is used to identify non-crystallographic translations that yield a high correlation of density. These rotation/translation pairs that yield high correlation (typically all those at or above 75 % of the highest value found) represent a possible set of NCS operators relating the various copies of the molecule in the asymmetric unit of the unit cell. Additionally, the candidate point used to identify NCS and the NCS-related points in the map may be considered approximate centers of regions where NCS applies. They are used below as the starting point for identification of the region where NCS operators apply.

### Evaluating the correlation of NCS-related density

Once a set of candidate NCS operators is obtained, the correlation of NCS-related density is examined in the original map, and the NCS operators are refined. This is done using the same approach as has been applied to NCS operators obtained from heavy-atom positions [18]. First the shape and position of the local region that is repeated by NCS (the asymmetric unit of NCS) is found. Then the NCS operators are refined to maximize the correlation of density among these NCS-related regions. The region where NCS applies is assumed to contain the point identified in the first step of our procedure that is within the macromolecule. A region with arbitrary shape surrounding this point is then chosen by sequential addition of new boundary points such that the local correlation of NCS-related density at each point in the region is above a threshold (typically 1/4 of the maximum correlation in the region). The local correlation of density [21] is calculated using map grid points within a sphere (normally with the radius used for identification of the solvent boundary, Terwilliger 2000b), centered at the point in question.

If the final correlation of NCS-related density (averaged over all pairs of NCS-related points) is above a threshold (typically 0.4) then the NCS operators are considered to represent actual NCS in the crystal and are used in later stages of structure determination. In cases where the starting map is very poor, this threshold may be decreased, increasing the sensitivity of the procedure but also increasing the probability of finding NCS where none is present.

## Results and discussion

**Fig. 1:**
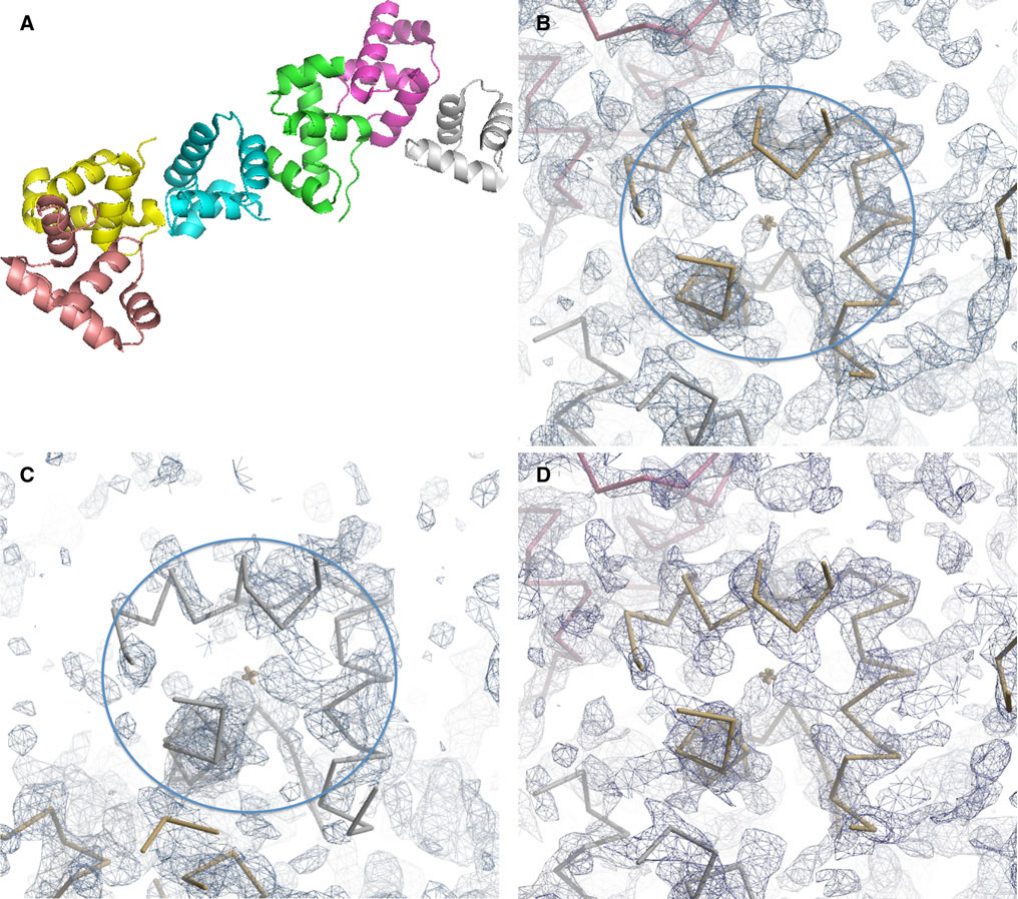
**a** Ribbon view of 6 chains of *gerE* in crystal structure PDB entry 1FSE [7]. **b** View of experimental MAD-phased, non density-modified electron density at a resolution of 2.7 Å near chain C of *gerE.* Electron density map calculated during automated structure solution with *phenix.autosol* [19]. The high density-variation location identified by *phenix.guess_molecular_centers* is marked, and *circle* with radius approximately 10 Å centered at this point illustrates the region that is to be cut out and compared to other density in the map. Chain C of *gerE* is shown in *yellow*. Contours for **b**–**d** are at 1.5σ. **c** As in **b**, except view is near chain F of *gerE,* shown in grey. **d** View as in **b** and **c**, except that the averaged density based on all 6 NCS-related copies is shown. **a** Created with *Pymol* [6]; **b**–**d** Created with *Coot* [8]

We applied our procedure to finding non-crystallographic symmetry to a structure where an electron density map of moderate quality could be obtained using MAD phasing. This structure (*gerE*, PDB entry 1FSE, [7]) consists of 6 copies of a protein chain with 74 residues. Figure 1a shows a schematic of the protein structure. It has 6 chains without any point-group symmetry. Figure 1b, c illustrates the MAD-phased electron density map for this structure for corresponding views of chains C and F, respectively, without any density modification or including any information on NCS. The deposited structure of *gerE* is shown as well. It may be seen that this map is of moderate quality in the region of chain C and rather poorer quality in the region of chain F.

The *Phenix* [1] tool *phenix.find_ncs_from*-*_density* was used to carry out the NCS identification. The density map was examined to find locations where the local variation of density was highest using the method *phenix.guess_molecular_centers*. The location with the highest local variation is marked in Fig. 1b. It can be seen that this point is well within the region of the map with protein-like characteristics (relatively connected and high density). This point is near the center of chain C of *gerE.*

Next, after truncating the resolution of the map to 4 Å, test density corresponding to a sphere of radius 10 Å of density centered at the point marked in Fig. 1b was cut out (the region inside the circle in Fig. 1b, except for the change in resolution of the map). The density within all 10 Å spherical regions in the 4 Å map was then compared to this test density using an FFT-based method, sampling all possible rotations of the test density on a grid. This density correlation procedure correctly identified all 5 regions that had high similarity to the test density. Figure 1c shows one of these related regions of density, the one corresponding to chain F of *gerE,* in the same view as Fig. 1b. It may be seen that within the 10 Å sphere the density in Fig. 1b, c are similar, though the density near chain F (Fig. 1c) is considerably less clear. Outside this region the shape of the density becomes less similar, as expected because the protein chains surrounding the chain located outside the sphere in Fig. 1b have a different arrangement than those outside the sphere in Fig. 1c.

The rotation and translation operators used to superimpose the density in Fig. 1b with that in Fig. 1c and the other 4 locations within the asymmetric unit of the density in this crystal were then refined to identify the region over which correlation of density existed and to optimize the correlation of density among all copies of this repeated density. This process yields as well the transformation matrices describing the relationships among all the NCS-related copies in the asymmetric unit of the crystal. The final average pair-wise correlation of density among the 6 copies was 0.53. This varied considerably among the 6 copies. For chain C (Fig. 1b) the average correlation to other NCS-related regions was 0.56, while for chain F (Fig. 1c), it was only 0.42. Figure 1d shows the average density for all the regions of density that were found to be similar from the same viewpoint as shown in Fig. 1b. It can be seen that this density has features even more like those expected of a density map of a protein than the individual regions of density (the tubes of density are better-connected).

## Conclusions

The approach described here for finding NCS in a map can be used in a number of situations. In the *Phenix* crystallographic software [1], the method *phenix.find_ncs_from_density* is used during structure solution with *phenix.autosol* [19] if NCS is expected but cannot be found from heavy-atom sites, and it is used during iterative model-building with *phenix.autobuild* [20] any time it is expected and has not been identified by any other means. The NCS relationships identified in this way can be used as a powerful addition to the information available for density modification [4, 12–14]. These relationships can also be used in model-building, where an incompletely built chain can be built using a more complete NCS-related copy of the same chain as a template [9, 15, 20]. Further they could potentially be used to place copies of entire chains in low-resolution maps.

The maps to be analyzed for NCS can be crystallographic maps, maps from electron microscopy, or maps from other sources. The approach described here could also be used to find density that is similar in two different electron density maps. This last application could be useful in automatically finding relationships between molecules in different crystals when carrying out cross-crystal averaging.
